# Risky Gambling Behaviors: Associations with Mental Health and a History of Adverse Childhood Experiences (ACEs)

**DOI:** 10.1007/s10899-021-10040-3

**Published:** 2021-06-23

**Authors:** Lindsay A. Bristow, Tracie O. Afifi, Samantha Salmon, Laurence Y. Katz

**Affiliations:** 1 Max Rady College of Medicine, University of Manitoba, 727 McDermot Ave, Winnipeg, MB R3E 3P5 Canada; 2 Departments of Community Health Sciences and Psychiatry, University of Manitoba, S113-750 Bannatyne Avenue, Winnipeg, MB R3E 0W5 Canada; 3 Department of Community Health Sciences, University of Manitoba, 750 Bannatyne Ave, Winnipeg, MB R3E 0W3 Canada; 4 Department of Psychiatry, University of Manitoba, PZ-162, 771 Bannatyne Ave, Winnipeg, MB R3E 3N4 Canada

**Keywords:** Adverse childhood experiences, ACEs, Gambling, Mental health, Well-being, Childhood adversity

## Abstract

Problem gambling and adverse childhood experiences (ACEs) are highly co-morbid and lead to numerous adverse health outcomes. Research demonstrates that greater levels of well-being protect individuals from experiencing ACE-related harms after a history of childhood adversity; however, this relationship has not been examined in the gambling literature. We hypothesized that individuals who experienced ACEs would engage in more problem gambling behaviors. We also hypothesized that individuals who experienced ACEs and reported flourishing mental health would have lower rates of problem gambling than individuals who experienced ACEs but did not report flourishing mental health. We conducted a secondary data analysis of the adult sample in the Well-Being and Experiences (WE) Study. Examining a parent population, parents and caregivers (*N* = 1000; *M*_age_ = 45.2 years; 86.5% female) of adolescents were interviewed on a variety of measures, including their history of ACEs, their gambling behaviors within the past year, and their mental health and well-being. We used multinomial logistic regression analysis to examine the relationship between 15 ACEs and gambling type (i.e., non-gambler, non-problem gambler, at-risk/problem gambler). We used interaction terms between each ACE and mental health to examine the moderating role of flourishing mental health and well-being. ACEs were associated with at-risk/problem gambling supporting hypothesis 1. Contrary to hypothesis 2, overall, flourishing mental health did not moderate the relationship between ACEs and gambling severity except for one ACE. In this study, we were able to gain a better understanding of how different ACEs each contribute to varying levels of gambling severity.

## Introduction

Problem gambling is a public health concern (Chou & Afifi, 2011; Faregh & Derevensky, 2013), with a population prevalence of 2.4% in Canada (Williams et al., 2012). Problem gambling, a subclinical form of gambling disorder, is defined as “gambling behavior that creates negative consequences for the gambler, others in his or her social network, or for the community” (Ferris & Wynne, 2001, p. 8). In its more severe form, gambling disorder is a psychiatric diagnosis including set diagnostic criteria and is characterized by “persistent and recurrent problematic gambling behavior leading to clinically significant impairment or distress” (American Psychiatric Association, 2013, p. 585). At-risk gamblers, the least severe along the spectrum, endorse fewer problem gambling correlates and have not yet necessarily experienced negative consequences resulting from their gambling behaviors (Ferris & Wynne, 2001).

Problem gambling is associated with many comorbid disorders and adverse outcomes, including mood disorders (Afifi et al., 2016; Kennedy et al., 2010; Kessler et al., 2008; Lorains et al., 2011; Martin et al., 2014; Quigley et al., 2015), anxiety disorders (Afifi et al., 2016; Kessler et al., 2008; Lorains et al., 2011), and substance use disorders (Afifi et al., 2016; Kessler et al., 2008; Lorains et al., 2011; Tackett et al., 2017). Additionally, gambling problems are associated with an increased prevalence of hypertension (Subramaniam et al., 2015), migraines (Afifi et al., 2010b), and obesity (Black et al., 2013; Morasco et al., 2006). Given the high prevalence of problem gambling and associated harms, there has been substantial research on risk factors for their development (Dowling et al., 2017; Quigley et al., 2015).

More recently, a history of adverse childhood experiences (ACEs) has been associated with problem gambling (Lotzin et al., 2018). ACEs have been extensively researched as risk factors for numerous adverse health consequences on multiple organ systems (Anda et al., 2008; Gilbert et al., 2015; Ross et al., 2020). Further, ACEs have been consistently associated with depressed affect (Afifi et al., 2006, 2014; Atzl et al., 2019; Negriff, 2020), suicide attempts (Afifi et al., 2014, 2017; Duke et al., 2010), anxiety (Afifi et al., 2006, 2014; Negriff, 2020)**,** post-traumatic stress disorder (Afifi et al., 2014; Atzl et al., 2019; Dvir et al., 2014), and substance use (Afifi et al., 2006, 2012, 2020b; Dube et al., 2006; Moss et al., 2020). More broadly, ACEs have also been associated with a range of behavioral addictions including food addiction and binge eating (Imperatori et al., 2016), internet addiction (Arslan, 2017; Dalbudak et al., 2014), and sex addiction (Kotera & Rhodes, 2019). ACEs are highly prevalent in North America, with many individuals reporting experiencing at least one ACE (69.1–75.1%; Afifi et al., 2020b; Poole et al., 2017, 2018) and, commonly, people report experiencing up to four or more ACEs (5.9–20.7%; Cronholm et al., 2015; Dong et al., 2004; Dube et al., 2003; Poole et al., 2018).

Since first being described by Felitti et al. (1998) over 20 years ago, ACEs have been classically categorized as forms of child maltreatment (i.e., emotional, physical, and sexual abuse; emotional and physical neglect) and forms of household challenges (i.e., exposure to intimate partner violence [IPV], household substance abuse, household mental illness, parental separation or divorce, and parental trouble with police/incarceration). More recently, studies have provided evidence for the inclusion of five expanded ACEs: spanking, child protective organization (CPO) contact, poverty, parental gambling, and peer victimization (Afifi et al., 2017, 2020a, 2020b). Previous research has indicated that childhood physical abuse is associated with increased odds of problem gambling (Afifi et al., 2010a). Other research has identified an association of problem gambling with other forms of child maltreatment (i.e., emotional abuse, sexual abuse, emotional neglect, and physical neglect; Black et al., 2012; Lane et al., 2016; Poole et al., 2017; Sharma & Sacco, 2015). However, there is little research on the role of household challenge-specific ACEs as risk factors for the development of problem gambling during adulthood (Lotzin et al., 2018; Poole et al., 2017). Furthermore, most research on household challenge-specific ACEs has been on disordered gambling (Lotzin et al., 2018; Poole et al., 2017) and has not considered the continuum of gambling behaviors (i.e., non-gambler, non-problem gambler, at-risk gambler). Finally, while some research has examined whether parental gambling is associated with the intergenerational transmission of problem gambling (Donati et al., 2013; Dowling et al., 2018; Hodgins et al., 2012), there is an absence of research examining the relationships between the other new expanded ACEs and gambling problems.

What has not been studied to date is whether mental health and well-being may mitigate the trajectory from ACEs to problem gambling. Well-being is a multi-dimensional construct, including emotional, psychological, and social domains (Lamers et al., 2009) and refers to the degree an individual experiences life satisfaction, positive affect, and self-efficacy (Diener, 2009). Individuals with higher levels of well-being are more likely to engage in emotion regulatory strategies, such as reappraisal (Banyard et al., 2017; Gross & John, 2003), and have more positive appraisals regarding their life circumstances (Diener, 2009). In contrast, lower levels of well-being are associated with maladaptive cognitions and coping styles (KL et al., 2017), which may lead some people to gamble in an effort to distract themselves from distressing emotions (Blaszczynski & Nower, 2002; Bristow et al., 2018; Moon et al., 2017). Researchers have consistently demonstrated that greater levels of well-being protect individuals from experiencing ACE-related harms (e.g., poor psychological and physical health) after a history of childhood adversity (Baiden et al., 2016; Banyard et al., 2017; Crandall et al., 2019). Research has also demonstrated that various socioemotional states in adulthood can have indirect protective effects between ACEs and related harms (Brett et al., 2018; Howell et al., 2020). It is, therefore, reasonable to suspect that mental health and well-being may also temper the relationship between ACEs and problem gambling; however, this has yet to be examined.

Based on the current gaps in the literature, we examined each ACE in relation to gambling problems and their contributions to gambling severity (i.e., non-gambler, non-problem gambler, at-risk gambler). We hypothesized that individuals who had experienced ACEs compared to those who had not would be more likely to engage in problem gambling behaviors. We also hypothesized that flourishing mental health and well-being would be protective and moderate the relationship between ACEs and gambling problems. Specifically, we hypothesized that individuals who experienced ACEs but who also reported flourishing mental health would have lower rates of problem gambling than individuals who experienced ACEs but did not report flourishing mental health.

## Method

### Data and Sample

This study utilized secondary data from the larger Well-Being and Experiences (WE) Study, a primary quantitative data collection survey in Winnipeg, Canada and surrounding rural areas (Afifi et al., 2020a, 2020b). The study included a knowledgeable parent or primary caregiver (predominantly mothers) and an adolescent aged 14–17 years old. Respondents were contacted using random digit dialing in addition to convenience sampling methods (snowball sampling, responding to research advertisements) to connect with parents of adolescents aged 14–17 years old. Once a parent was identified as having a child between the ages of 14–17 years old and considered knowledgeable about the child, the parent was given information about the study and asked if he/she would be interested in participating in the study. Data were collected from July 2016 to October 2017. Only data from the parents/caregivers were used for this study (*N* = 1000). After obtaining informed consent, parents/caregivers were interviewed on a variety of measures, including their history of ACEs, their gambling behaviors within the past year, and their mental health and well-being. For a more comprehensive explanation of the sampling procedure and methods of the WE Study, see Afifi et al. (2020a, 2020b). Ethics approval was provided from the Health Research Ethics Board at the University of Manitoba.

### Measures

*Canadian Problem Gambling Index (CPGI)* Problem gambling was examined using an adapted version of the CPGI (Ferris & Wynne, 2001) with ten questions assessing possible gambling-related harms experienced within the past 12 months. Respondents answered each item on a 4-point scale (0 = never, 1 = sometimes, 2 = most of the time, 3 = almost always). Example items include “Have you bet more than you could really afford to lose?” and “Have you needed to gamble with larger amounts of money to get the same feeling of excitement?” The tenth item added to the CPGI was “In the past 12 months, have you gambled as a way of forgetting problems or to feel better when you were depressed?”. Responses are summed, and gamblers are divided into the following subtypes: non-problem, low-risk, moderate risk, problem gambler. Past-year gamblers who answered “never” to each question are considered non-problem gamblers. Those who did not gamble within the past 12 months were classified as non-gamblers. The CPGI has shown good internal consistency (α = 0.84; Ferris & Wynne, 2001). The present study had excellent internal consistency (α = 0.92). Due to insufficient power for the problem gambler group (< 2%), it was necessary to collapse the risk groups into at-risk/problem gamblers. Collapsing the risk groups is acceptable as researchers have found negligible differences to distinguish low-risk from moderate-risk groups (Currie et al., 2013) and has been used as a valid measurement in past studies (Afifi et al., 2016). Finally, because characteristics between non-gamblers and non-problem gamblers can differ considerably (Ferris & Wynne, 2001), these groups were examined separately.

*Adverse childhood experiences (ACEs)* A total of 15 ACEs were examined independently as well as in the following categories: child maltreatment (i.e., emotional abuse, physical abuse, sexual abuse, emotional neglect, physical neglect, and spanking), household challenges (i.e., exposure to physical IPV, household substance abuse, household mental illness, parental separation or divorce, parental trouble with police, CPO contact, poverty, and parental gambling), and peer victimization. This categorization follows the results of a recent factor analysis (Afifi et al., 2020a), which examined the empirical fit of these ACEs in child maltreatment or household challenges categories. However, we decided to separate peer victimization from child maltreatment since these experiences occur in different socialization domains (i.e., peers and family, respectively).

The Childhood Trauma Questionnaire (CTQ; Bernstein & Fink, 1998) measured physical abuse, sexual abuse, emotional abuse, physical neglect, and emotional neglect, each with five items asking about the respondent’s experiences growing up. Items were dichotomized using cut-points based on CTQ coding guidelines. Adapted items from the Childhood Experience of Violence Questionnaire (CEVQ; Walsh et al., 2008) were used to measure exposure to physical IPV (i.e., asking if, before age 16, the participant had ever heard a parent/stepparent/guardian hit another adult in the home) and spanking (i.e., asking if, before age 11, how frequently they recalled being spanked in a regular year). Items were dichotomized using recommended cut-points (Walsh et al., 2008). Adapted yes/no items from the original ACEs Study (Felitti et al., 1998) were used to assess household substance abuse, household mental illness, parental separation or divorce, and parental trouble with police. All measures were related to the participant’s experiences before age 16 years old. Household substance abuse was assessed with two items, and the other three ACEs were each assessed with one item.

Furthermore, questions were created to measure four of the expanded ACEs: peer victimization, parental gambling, CPO contact, and poverty. All questions asked participants to answer about their experiences before age 16 years old, and each item was dichotomized. Peer victimization was assessed with two items and was coded yes if verbal peer victimization occurred more than 10 times and/or if physical bullying occurred 3 or more times. An example item is “Sometimes kids get hassled or picked on by other kids who say hurtful or mean things to them. Before the age of 16 years old, how many times did this happen to you?” Parental gambling was assessed with one yes/no item about whether any adults in the home had gambling problems. Similarly, CPO contact was assessed with one yes/no item. CPO contact included any of the following: social services, child welfare, children's aid, or the Ministry. Finally, poverty was assessed with two items that asked about whether the family had ever experienced financial difficulty paying for the rent/mortgage or basic necessities (e.g., food, clothing). Poverty was coded yes if either item occurred “sometimes” or more often (1 = never, 2 = rarely, 3 = sometimes, 4 = often, 5 = very often).

*Mental Health Continuum Short Form (MHC-SF)* The MHC-SF (Lamers et al., 2009) measures well-being across three domains (emotional well-being, psychological well-being, and social well-being) with a total of 14 items. The emotional well-being subscale consists of three items (happiness, interest in life, and life satisfaction). An example item is “During the past month, how often did you feel interested in life?” Psychological well-being and social well-being are both defined as aspects of positive functioning and consist of six items and five items, respectively. An example item from the psychological well-being subscale is “During the past month, how often did you feel confident to think or express your own ideas and opinions?” An example item from the social well-being subscale is “During the past month, how often did you feel that you belonged to a community (like a social group, your school, or your neighborhood)?” Individuals report the frequency they experience each item (“every day,” “almost every day,” “once or twice,” “never”). When created, the scale was coded as follows: respondents who experience a minimum of one item from the emotional domain and a combined six items from psychological/social well-being on a daily/bi-daily basis are considered to have flourishing mental health. If the respondent answers that they experience at least one item from the emotional domain and a combined six items from the other two domains twice or less a month, then they are coded as having languishing mental health. If the respondents did not meet either criteria, they were coded to have moderate mental health. The MHC-SF has shown good internal consistency (α > 0.80; Lamers et al., 2009). The present study had excellent internal consistency (α = 0.93). Since very few individuals were languishing, and flourishing mental health was used as our moderator, this variable was dichotomized to flourishing vs. non-flourishing (i.e., moderate or languishing) mental health.

*Covariates* Sex (i.e., female, male), age, ethnicity (i.e., white only, other/multi-ethnicity), birth country (i.e., Canada, another country), highest level of education (i.e., high school completion or less, some post-secondary, completed trade school or community college, completed a university undergraduate degree, completed a university graduate degree), employment status (i.e., employed, other), household income (i.e., $49,999 or less, $50,000–$99,999, $100,000–$149,999, $150,000 or more, no response), and marital status (i.e., married or common-law, separated/divorced/widowed, never married) were all used as covariates.

### Statistical Analysis

First, sociodemographic characteristics among the total sample and by gambling type (i.e., non-gambler, non-problem gambler, and at-risk/problem gambler) were computed. Differences were examined with chi-square tests for categorical variables and one-way ANOVA for continuous variables. Second, the prevalence of ACEs and mental health and well-being by gambling type was computed. Third, multinomial logistic regression analysis was used to examine the relationship between ACEs and gambling type, first unadjusted and subsequently adjusting for sociodemographic characteristics. Finally, interaction terms between each ACE and mental health and well-being were included to examine the moderating role of flourishing mental health and well-being. For all calculations, non-gamblers were used as the reference category. All analyses were conducted at the 0.05 level of significance using Stata version 15.1.

## Results

Sociodemographic characteristics are located in Table 1. Among the sample (86.5% female, mean age = 45.2 years), 43.4% of respondents were non-gamblers, 46.6% were non-problem gamblers, and 10.2% were at-risk/problem gamblers. Significant differences among gambling types were noted for all sociodemographic factors except age.

**Table 1:** Sociodemographic characteristics of the sample by gambling type

Characteristic	Total Sample (N = 1000)	Non-Gambler (43.0%)	Non-Problem Gambler (46.8%)	At-risk/Problem Gambler (10.2%)	*p-*value*
*Sex, %*					
Female	86.5	90.4	83.3	84.2	.007
Male	13.5	9.6	16.7	15.8	
Age, mean (SD)	45.2 (6.0)	45.4 (5.9)	45.2 (6.1)	44.3 (6.5)	.223
*Country of Birth, %*					
Canada	75.9	66.8	83.6	80.2	< .001
Another country	24.1	33.3	16.4	19.8	
*Education, %*					
High school completion or less	14.1	11.0	13.6	28.7	< .001
Some post-secondary	11.6	9.9	14.0	7.9	
Completed trade school or community college	24.2	20.4	26.8	27.7	
Completed a university undergraduate degree	24.9	28.4	23.3	18.8	
Completed a university graduate degree	25.2	30.3	22.3	16.8	
*Employment Status, %*					
Employed	83.9	81.3	87.6	78.2	.011
Other	16.1	18.7	12.5	21.8	
*Household Income, %*					
$49,999 or less	19.9	22.8	14.7	31.7	< .001
$50,000 to $99,999	35.1	37.1	32.4	39.6	
$100,000 to $149,999	22.2	17.1	27.9	18.8	
$150,000 or more	18.0	18.5	19.9	6.9	
No response	4.8	4.5	5.2	3.0	
*Marital Status, %*					
Married or common-law	79.2	80.9	82.1	58.0	< .001
Separated, divorced, widowed	15.2	15.5	13.6	23.0	
Never married	5.6	3.5	4.3	19.0	

SD = standard deviation

*χ^2^ tests were used for categorical variables and a one-way ANOVA test was used for age

Descriptive statistics for the percent of ACEs experienced among each gambling type are located in Table 2. Table 2 also includes information about the groups’ mental health and well-being. Non-gamblers and non-problem gamblers experienced similar prevalence rates for flourishing mental health (68.2% vs. 67.1%). In comparison, flourishing mental health among at-risk/problem gamblers was markedly lower (47.4%).

**Table 2:** Prevalence of adverse childhood experiences (ACEs) and mental health and well-being by gambling type

ACE	Total Sample (N = 1000)	Non-Gambler (43.0%)	Non-Problem Gambler (46.8%)	At-risk/ Problem Gambler (10.2%)
*Child Maltreatment ACE*				
Physical abuse, %	22.2	19.6	21.3	36.0
Sexual abuse, %	27.0	24.6	27.3	35.7
Emotional abuse, %	17.0	15.7	16.1	24.2
Physical neglect, %	25.3	25.4	22.6	35.6
Emotional neglect, %	14.0	15.3	10.8	22.8
Spanking, %	45.3	45.8	41.5	60.0
Any child maltreatment ACE, %	66.0	67.1	61.1	82.2
*Household Challenges ACE*				
Exposure to physical IPV, %	12.6	11.9	10.9	22.2
Household substance abuse, %	30.4	29.8	28.3	42.0
Household mental illness, %	32.7	31.8	32.1	37.8
Parental separation/divorce, %	23.5	19.9	24.6	31.5
Parental trouble with police, %	8.6	5.8	10.0	13.4
Parental gambling, %	6.3	4.3	5.8	16.3
CPO contact, %	7.4	5.0	8.5	12.9
Poverty, %	40.0	36.0	39.5	57.1
Any household challenge ACE, %	70.8	67.2	70.4	85.3
*Peer Victimization ACE*				
Peer Victimization, %	45.6	42.7	45.8	56.3
Mental Health and Well-being, %*				
Flourishing	65.6	68.2	67.1	47.4
Non-flourishing (moderate/languishing)	34.4	31.8	32.9	52.6

*IPV * intimate partner violence,* CPO * child protective organization

*X^2^ = 15.5, *df* = 2, *p* < .001

In Table 3, except for emotional neglect, all unadjusted odds ratios (ORs) for the other child maltreatment ACEs (i.e., physical abuse, sexual abuse, emotional abuse, physical neglect, spanking, any child maltreatment ACE, and peer victimization) showed significant associations indicating an increased likelihood of being an at-risk/problem gambler compared to non-gambler. This relationship remained significant when adjusting for covariates for physical abuse (adjusted OR [AOR] = 1.92; 95% CI = 1.15–3.19), spanking (AOR = 1.76; 95% CI = 1.09–2.84), and any child maltreatment ACE (AOR = 2.06; 95% CI = 1.15–3.70). In the unadjusted model, emotional neglect significantly decreased the odds of being a non-problem gambler compared to non-gambler but was no longer significant when adjusting for covariates. There were no significant interaction effects for any child maltreatment ACEs or peer victimization with mental health and well-being for any gambling type.

**Table 3:** Associations between adverse childhood experiences (ACEs) and gambling type, and the moderating effect of flourishing mental health and well-being

ACE	Non-Problem Gambler (versus Non-Gambler)			At-risk/Problem Gambler (versus Non-Gambler)		
	OR (95% CI)	AOR (95% CI)	Interaction *p*-value	OR (95% CI)	AOR (95% CI)	Interaction *p*-value
*Child Maltreatment ACE*						
Physical abuse	1.11 (0.80–1.54)	1.12 (0.79–1.60)	.533	2.30 (1.43–3.70)**	1.92 (1.15–3.19)*	.258
Sexual abuse	1.15 (0.85–1.56)	1.07 (0.77–1.49)	.955	1.70 (1.06–2.72)*	1.30 (0.78–2.17)	.227
Emotional abuse	1.03 (0.72–1.48)	1.02 (0.69–1.52)	.915	1.72 (1.01–2.92)*	1.31 (0.73–2.35)	.739
Physical neglect	0.86 (0.63–1.17)	0.87 (0.62–1.22)	.301	1.63 (1.03–2.59)*	1.31 (0.79–2.19)	.561
Emotional neglect	0.67 (0.45–0.99)*	0.68 (0.44–1.03)	.225	1.63 (0.95–2.79)	1.17 (0.65–2.11)	.984
Spanking	0.84 (0.64–1.10)	0.83 (0.62–1.11)	.320	1.78 (1.14–2.77)*	1.76 (1.09–2.84)*	.487
Any child maltreatment ACE	0.77 (0.58–1.02)	0.79 (0.59–1.07)	.937	2.26 (1.31–3.92)**	2.06 (1.15–3.70)*	.933
*Peer Victimization ACE*						
Peer Victimization	1.14 (0.87–1.49)	0.94 (0.71–1.26)	.447	1.73 (1.10–2.71)*	1.52 (0.93–2.48)	.327

*OR * odds ratio,* CI*  confidence interval,* AOR * odds ratio adjusted for sociodemographic characteristics

*p < .05

**p < .01

***p < .001

In Table 4, except for household mental illness, all unadjusted models for the other household challenge ACEs (i.e., exposure to physical IPV, household substance abuse, parental separation/divorce, parental trouble with police, parental gambling, CPO contact, poverty, and any household challenge ACE) showed significant associations indicating an increased likelihood of being an at-risk/problem gambler compared to non-gambler. Overall, the highest odds ratios for being an at-risk/problem gambler compared to non-gambler were parental gambling, any household challenge, CPO contact, and parental trouble with police. After adjusting for covariates, this relationship remained significant for parental gambling (AOR = 3.37; 95% CI = 1.53–7.43), poverty (AOR = 2.19; 95% CI = 1.34–3.60), and any household challenge ACE (AOR = 2.03; 95% CI = 1.07–3.85). Furthermore, parental trouble with police and CPO contact were found to significantly increase the odds of being a non-problem gambler compared to non-gambler. Household mental illness did not significantly increase the likelihood of being a non-problem gambler or at-risk/problem gambler when compared to non-gamblers. A significant interaction effect was found between exposure to physical IPV and flourishing mental health. Findings indicated that having flourishing mental health and well-being was protective against gambling problems for those without a history of exposure to IPV, but this was not the case for those with a history of exposure to IPV.

**Table 4:** Associations between adverse childhood experiences (ACEs) and gambling type, and the moderating effect of flourishing mental health and well-being

ACE	Non-Problem Gambler (versus Non-Gambler)			At-risk/Problem Gambler (versus Non-Gambler)		
	OR (95% CI)	AOR (95% CI)	Interaction *p*-value	OR (95% CI)	AOR (95% CI)	Interaction *p*-value
*Household Challenges ACE*						
Exposure to physical IPV	0.91 (0.60–1.38)	0.91 (0.58–1.43)	.436	2.11 (1.21–3.70)**	1.55 (0.83–2.91)	.041
Household substance abuse	0.93 (0.69–1.24)	0.87 (0.63–1.19)	.654	1.71 (1.09–2.67)*	1.37 (0.84–2.24)	.405
Household mental illness	1.02 (0.75–1.37)	0.89 (0.64–1.23)	.670	1.30 (0.81–2.10)	0.92 (0.54–1.57)	.667
Parental separation/divorce	1.32 (0.95–1.82)	1.14 (0.80–1.62)	.084	1.86 (1.12–3.07)*	1.35 (0.78–2.35)	.121
Parental trouble with police	1.82 (1.09–3.04)*	1.44 (0.83–2.50)	.213	2.53 (1.24–5.17)*	1.27 (0.56–2.85)	.070
Parental gambling	1.37 (0.74–2.53)	1.49 (0.77–2.87)	.727	4.34 (2.12–8.86)***	3.37 (1.53–7.43)**	.806
CPO contact	1.77 (1.02–3.06)*	1.44 (0.79–2.61)	.346	2.82 (1.36–5.85)**	1.46 (0.65–3.31)	.399
Poverty	1.16 (0.88–1.54)	1.30 (0.95–1.78)	.942	2.37 (1.51–3.71)***	2.19 (1.34–3.60)**	.586
Any household challenge ACE	1.16 (0.86–1.56)	1.15 (0.83–1.58)	.066	2.83 (1.55–5.18)**	2.03 (1.07–3.85)*	.246

*IPV * intimate partner violence,* CPO*  child protective organization,* OR*  odds ratio,* CI* confidence interval,* AOR*  odds ratio adjusted for sociodemographic characteristics

*p < .05

**p < .01

***p < .001

## Discussion

The primary aim of this study was to examine the relationship of ACEs to gambling behaviors and to understand whether flourishing mental health would be associated with decreased gambling severity in those with a history of ACEs. The results of the present study corroborate past research on the association between ACEs and at-risk/problem gambling (Afifi et al., 2010a; Black et al., 2012; Lane et al., 2016; Lotzin et al., 2018; Poole et al., 2017; Sharma & Sacco, 2015). This study also extends the existing literature by examining the newly proposed expanded ACEs (Afifi et al., 2017, 2020a, 2020b) and their relationships to gambling behaviors and gambling severity. While it is not possible to infer causality, these results provide novel information regarding the strength of associations between distinct forms of childhood adversity and different gambling outcomes.

Except for emotional neglect and household mental illness, all other ACEs significantly increased the likelihood of being an at-risk/problem gambler (when compared to non-gambler). Parental trouble with police and CPO contact also significantly increased the odds of being a non-problem gambler (compared to non-gambler). Contrary to previous research (Poole et al., 2017), household mental illness did not significantly increase the odds of any gambling behavior. Similarly, emotional neglect did not increase the odds of gambling behavior but instead decreased the likelihood of being a non-problem gambler (compared to non-gambler). This result is not surprising as there have been inconsistent findings regarding whether emotional neglect significantly increases the likelihood of addictive behaviors such as problem gambling (Black et al., 2012; Poole et al., 2017) or substance use (Afifi et al., 2020b; Merrick et al., 2017; Mersky et al., 2017). Of the original 10 ACEs, except for physical abuse and any non-specific child maltreatment ACE or any non-specific household challenge ACE, these associations were no longer significant after adjusting for covariates.

While there is limited research that has studied the newly proposed expanded ACEs and documented their association with substance use (Afifi et al., 2017, 2020b; Merrick et al., 2017; Mersky et al., 2017), this is the first time these ACEs have been examined within the context of the gambling literature. All expanded ACEs (i.e., spanking, peer victimization, parental gambling, CPO contact, and poverty) significantly increased the odds of being an at-risk/problem gambler (compared to non-gambler). Further, we observed that three of the five new ACEs (i.e., spanking, parental gambling, and poverty) remained significant after adjusting for sociodemographic factors and they were among the highest odds ratios for an increased likelihood of at-risk/problem gambling (compared to non-gambling).

As expected, and consistent with past literature (Afifi et al., 2016; Currie et al., 2013), we observed that a lower proportion of at-risk/problem gamblers experienced flourishing mental health when compared to non-gamblers and non-problem gamblers. The plots of the significant interaction effect indicated that a greater proportion of individuals reporting flourishing mental health after exposure to physical IPV were at-risk/problem gamblers (compared to non-gamblers and non-problem gamblers). Contrary to our hypothesis, flourishing mental health and well-being was not observed to moderate the relationship between ACEs and gambling severity in the way that was expected. Rather, we found that flourishing mental health and well-being did reduce problem gambling among those without a history of exposure to IPV but did not do this for those with a history of exposure to IPV. This finding is also contrary to extensive research demonstrating that greater levels of well-being, following a history of childhood adversity, ordinarily protect individuals from experiencing ACE-related harms (Baiden et al., 2016; Banyard et al., 2017; Crandall et al., 2019). Therefore, a future area of research could examine if well-being could become protective if treatments were available to improve mental health and well-being following exposure to physical IPV.

While some of these results appear counterintuitive, some studies have found that child maltreatment ACEs (i.e., physical, emotional, sexual abuse; physical and emotional neglect), as opposed to household challenge ACEs (e.g., exposure to IPV), lead to more socioemotional problems (Narayan et al., 2017) and adverse mental health outcomes (Atzl et al., 2019; Negriff, 2020). In one study, 80% of participants who had been exposed to IPV reported relatively no difficulty regulating their emotions as adults (Suzuki et al., 2008). Another study found that certain household challenge ACEs, including exposure to IPV, did not significantly predict psychological distress (Finkelhor et al., 2015) and therefore may negatively impact well-being to a lesser extent. Individuals engaging in at-risk/problem gambling after exposure to physical IPV may be doing so for other gambling motives (Milosevic & Ledgerwood, 2010) and not as a means to cope with distressing emotions. An additional explanation could be that well-being may simply not affect gambling severity. Research has shown that, in a clinical sample of recovering pathological gamblers, there were no significant differences in levels of well-being among individuals who later became non-problem gamblers or those who abstained completely (Muller et al., 2017).

This work has clinical implications. The ACE observed to most significantly increase the likelihood of at-risk/problem gambling was parental gambling. Some research has found that parental gambling is associated with increased gambling frequency in adulthood but does not significantly influence whether the individual is more likely to be a non-problem gambler or problem gambler (Hodgins et al., 2012). Our study demonstrates that parental gambling increases the likelihood of being an at-risk/problem gambler, but not a non-problem gambler (when compared to non-gamblers). These results, in part, may be due to the complex interaction that exists between early life stressors and an individual’s genetic vulnerability towards addiction (for reviews, see Buchanan & Lovallo, 2019, and Enoch, 2012). This finding is concerning as our sample consisted exclusively of parents suggesting that their children may also be at risk for developing future gambling problems (Donati et al., 2013; Dowling et al., 2018). With knowledge of the adverse consequences that result from problem gambling, these results emphasize the importance of gambling treatment programs targeted at parents. Clinicians should also be aware of this relationship to determine if children might be at-risk for gambling problems. Further work is needed to understand how to mitigate the risk best.

Our study had several limitations. First, our study had a small sample size which limited our power for running certain statistical models. The restricted sample size also necessitated that we consider the continuum of gambling risk (i.e., low-risk, moderate-risk, problem gamblers) as one variable which may have diluted the strength of associations. Second, our study used a non-representative sample that consisted solely of parents. Therefore, the study had a disproportionately high number of female respondents due to more mothers than fathers answering as the most knowledgeable person about their child. This gender disproportion may have weakened the observed associations between ACEs and gambling severity as research shows that men engage in more problem gambling behaviors (Faregh & Derevensky, 2013; Thomas et al., 2011). Future research could use a more gender-balanced sample to examine these relationships. In addition, specific characteristics of parenthood may make individuals more susceptible to engage in, or refrain from, risky gambling behaviors. The odds of developing gambling problems following a history of ACEs should be compared to samples of the general population and non-parent populations to explore these potential differences. Third, our study relied on self-report data. Researchers have found that gamblers engage in impression management when self-reporting on gambling problems (Kuentzel et al., 2008) which may have falsely over-inflated our proportion of non-problem gamblers. However, among all gamblers, the estimated population prevalence of non-problem gamblers in Canada is 85% (Currie et al., 2013). Of all individuals who reported any gambling behaviors in this study, the prevalence of non-problem gamblers was 82%, indicating there was likely little over-inflation in our study.

In addition, our study employed a cross-sectional design. Therefore, we are unable to establish a causal relationship between ACEs and at-risk/problem gambling during adulthood. There may have been several unmeasured confounders responsible for the strength of the observed associations between ACEs and gambling severity that were not accounted for in the study. Further, without establishing temporal precedence, it is not possible to exclude the possibility that gambling problems preceded low levels of well-being. It is well-documented that a complex bidirectional relationship exists between negative affective states and problem gambling (Kessler et al., 2008; Sundqvist & Rosendahl, 2019). To address these challenges, future research should employ a longitudinal design. Finally, the study relied on retrospective recall of an individual’s history of ACEs. While this form of recall can be subject to bias and inaccuracy, retrospective recall is commonly employed in ACEs research (Afifi et al., 2014, 2017; Atzl et al., 2019; Howell et al., 2020; Lu et al., 2008; Narayan et al., 2017).

In summary, this is the first study to elucidate the relationships between the new expanded ACEs (Afifi et al., 2017, 2020a, 2020b) and gambling problems. Our study provides evidence for the importance of studying these new ACEs as we have demonstrated that, in many instances, the new expanded ACEs have the most significant associations with gambling severity and retain their strength after adjusting for covariates. Past research on at-risk/problem gambling has also predominantly focused on child maltreatment ACEs (Afifi et al., 2010a; Black et al., 2012; Lane et al., 2016; Poole et al., 2017; Sharma & Sacco, 2015), largely ignoring household challenges. The prior studies examining household challenge ACEs (Lotzin et al., 2018; Poole et al., 2017) focused exclusively on disordered gamblers. In this study, we were able to examine these relationships across three gambling types (i.e., non-gambler, non-problem gambler, at-risk/problem gambler) to gain a better understanding of how these ACEs ultimately contribute to different levels of gambling severity.

## Data Availability

Data are not publicly available. The WE Study data were not anonymous. Due to sensitive nature of the data and privacy and confidentially guidelines, the data must be housed in a secured lab and cannot be made publicly available.
