# Correction to: Risky Gambling Behaviors: Associations with Mental Health and a History of Adverse Childhood Experiences (ACEs)

**DOI:** 10.1007/s10899-021-10063-w

**Published:** 2021-08-02

**Authors:** Lindsay A. Bristow, Tracie O. Afifi, Samantha Salmon, Laurence Y. Katz

**Affiliations:** 1 Max Rady College of Medicine, University of Manitoba, 727 McDermot Ave, Winnipeg, MB R3E 3P5 Canada; 2 Departments of Community Health Sciences and Psychiatry, University of Manitoba, S113‑750 Bannatyne Avenue, Winnipeg, MB R3E 0W5 Canada; 3 Department of Community Health Sciences, University of Manitoba, 750 Bannatyne Ave, Winnipeg, MB R3E 0W3 Canada; 4 Department of Psychiatry, University of Manitoba, PZ‑162, 771 Bannatyne Ave, Winnipeg, MB R3E 3N4 Canada

## Correction to: Journal of Gambling Studies 10.1007/s10899-021-10040-3

The article “Risky Gambling Behaviors: Associations with Mental Health and a History of Adverse Childhood Experiences (ACEs)”, written by Lindsay A. Bristow, Tracie O. Afifi, Samantha Salmon, Laurence Y. Katz, was originally published electronically on the publisher's internet portal on 23 June 2021 without open access. With the author(s)' decision to opt for Open Choice the copyright of the article changed on 7 July 2021 to © The Author(s) 2020 and the article is forthwith distributed under a Creative Commons Attribution 4.0 International License, which permits use, sharing, adaptation, distribution and reproduction in any medium or format, as long as you give appropriate credit to the original author(s) and the source, provide a link to the Creative Commons licence, and indicate if changes were made. The images or other third party material in this article are included in the article's Creative Commons licence, unless indicated otherwise in a credit line to the material. If material is not included in the article's Creative Commons licence and your intended use is not permitted by statutory regulation or exceeds the permitted use, you will need to obtain permission directly from the copyright holder. To view a copy of this licence, visit http://creativecommons.org/licenses/by/4.0.

 The original article has been corrected.

